# Does the Freeze–Thaw Technique Affect the Properties of the Alginate/Chitosan Glutamate Gels with Posaconazole as a Model Antifungal Drug?

**DOI:** 10.3390/ijms23126775

**Published:** 2022-06-17

**Authors:** Marta Szekalska, Katarzyna Sosnowska, Magdalena Wróblewska, Anna Basa, Katarzyna Winnicka

**Affiliations:** 1 Department of Pharmaceutical Technology, Medical University of Białystok, Mickiewicza 2c, 15-222 Białystok, Poland; katarzyna.sosnowska@umb.edu.pl (K.S.); magdalena.wroblewska@umb.edu.pl (M.W.); 2 Department of Physical Chemistry, Faculty of Chemistry, University of Białystok, Ciołkowskiego 1K, 15-245 Białystok, Poland; abasa@uwb.edu.pl

**Keywords:** sodium alginate, chitosan glutamate, freeze–thaw technique, posaconazole, hydrogels

## Abstract

Hydrogels are semi-solid systems with high flexibility, which, due to holding large amounts of water, are similar to natural tissues and are very useful in the field of biomedical applications. Despite the wide range of polymers available to form hydrogels, novel techniques utilized to obtain hydrogels with adequate properties are still being developed. The aim of this study was to evaluate the impact of the freeze–thaw technique on the properties of cryogels based on sodium alginate and chitosan glutamate with posaconazole as a model antifungal substance. The effect of the freezing and thawing process on the physicochemical, rheological, textural and bioadhesive properties of prepared cryogels was examined. Additionally, the antifungal activity against *Candida albicans*, *Candida parapsilosis* and *Candida krusei* of designed formulations was examined. It was shown that the freeze–thaw technique significantly improved viscosity, bioadhesiveness, textural properties and prolonged the in vitro posaconazole release. Moreover, alginate/chitosan glutamate cryogels exhibited higher values of inhibition zone in *C. parapsilosis* culture than traditional hydrogel formulations.

## 1. Introduction

Hydrogels constitute three-dimensional formulations with hydrophilic structures and are very useful in the field of drug delivery systems. Compared to other topical dosage forms, hydrogels, as a result of high water content, have physical properties similar to living tissue, suitable softness, flexibility and plasticity. They possess adequate mechanical strength and porous structure. Furthermore, by using natural polymers, features such as biocompatibility and biodegradability can be provided. Hydrogels might be used as carriers of both hydrophilic or lipophilic substances and might be applied orally, nasally, rectally, vaginally, ocularly or parenterally. However, due to their low mechanical strength and fragile nature, the utilization of hydrogels is still limited. Thus, hydrogels with stronger and more stable properties are still needed and various techniques for the preparation of hydrogels have been developed [1,2].

Sodium alginate (ALG) is a natural polymer obtained from seaweed or produced by bacteria. It has a number of advantages such as nontoxicity, biocompatibility and biodegradability. ALG belongs to the group of polysaccharides and is composed of β-D-mannuronic acid and α-L-guluronic acid residues joined by β (1–4) glycosidic linkages. ALG is characterized by unique properties including swelling, mucoadhesiveness and rapid gelling under mild conditions [3].

Chitosan (CS), similar to ALG, is a non-toxic, biocompatible and biodegradable polysaccharide received by deacetylation of chitin—the primary component of crustacean shells. It is composed of β-(1–4) linked D-glucosamine and N-acetyl-D-glucosamine randomly distributed [4]. CS possesses swelling and mucoadhesive properties. It belongs to the group of cationic mucoadhesive polymers and its amino groups interact with sialic acid, one of the components of mucin. Due to the CS low solubility at neutral pH, its utilization in the design of drug dosage forms is limited. Therefore, more soluble CS derivatives obtained, e.g., through the reaction of the amino- and hydroxyl groups with carboxy alkyl, hydroxy alkyl, and acyl derivatives are utilized. There are reports concerning the application of chitosan glutamate (CS-G) obtained from the connection of chitosan with glutamic acid, which is characterized by better mucoadhesive ability, water solubility and stronger antifungal activity than unmodified CS [5,6,7].

Fungal infections are one of the most common worldwide skin diseases, which may include dermatophyte, nondermatophyte and *Candida* infections. Skin candidiasis is generally caused by *Candida albicans,* and exceptionally by other species of this genus, e.g., *Candida parapsilosis*. Topical fungal infections can be either acute or chronic in nature and may develop as superficial candidiasis—restricted to specific parts of the body surface (skin and mucosa) or invasive candidiasis. Despite a wide range of available drugs, treatment of cutaneous fungal infections is cumbersome and long-lasting [8,9].

Posaconazole (POS) is a drug molecule from the triazole group, which is characterized by extended antifungal activity including *Candida* spp., *Cryptococcus* spp., *Aspergillus* spp., *Zygomycetes*, as well as endemic fungi and dermatophytes—*Trichophyton, Epidermophyton* and *Microsporum* spp. POS antifungal activity is associated with the inhibition of ergosterol—a component of fungal cytoplasmic membranes biosynthesis. POS is characterized by the low solubility in an aqueous environment, which affects its low bioavailability. Despite the well-documented POS activity against yeast and dermatophytes, topical dosage forms containing POS are not available [10,11,12].

The aim of this study was to develop ALG/CS-G hydrogels and cryogels with POS used as a model antifungal drug. To obtain cryogels, a physical technique—the freeze–thaw method—was utilized [13]. In the next step, the impact of the freezing and thawing process on the physicochemical, rheological, textural and bioadhesive properties of obtained formulations was performed. Moreover, the antifungal activity against selected *Candida* spp. (*C. albicans*, *C. parapsilosis* and *C. krusei*) of designed formulations was estimated.

## 2. Results and Discussion

ALG as a polyanion and CS as a polycation can create interactions between ALG carboxyl groups and CS amino groups. The combination of these polymers is characterized by better mechanical and thermal stability and higher adhesive properties than their constituent polymers [6,14,15].

**Figure 1:**
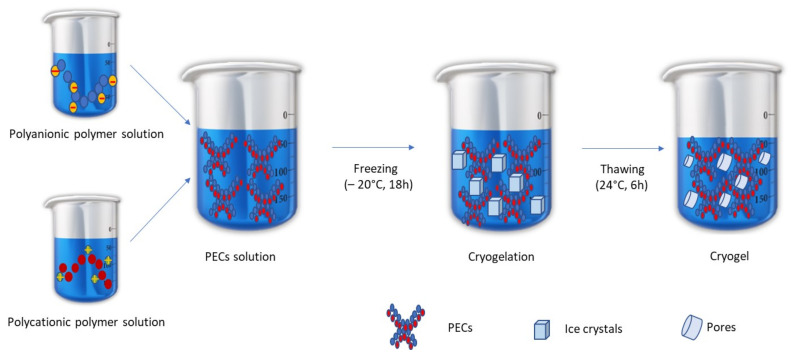
Scheme of ALG/CS-G cryogel preparation by the freeze–thaw method.

Cryogelation is a relatively new technique, which may be applied to prepare ALG hydrogels. This method is based on the non-deep freezing of a polymer solution, its storage in the frozen state, and the thawing process. During freezing, dissolved polymers concentrate and undergo interactions. Solvent crystallization occurs, which enables to minimize the space between polymer chains. After thawing, as a result of the creation of junction zones between uncharged polymers through van der Waals forces and hydrogen bonds, a cryogel is formed [13,16]. Figure 1 introduces the scheme of ALG/CS-G cryogel preparation by the freeze–thaw method.

### 2.1. Hydrogels and Cryogels Characteristics 

Hydrogel drug delivery systems possess many important advantages, such as non-greasy consistency, suitable softness, flexibility and plasticity, prolonged residence time at the application site and reduced administration frequency [17].

It was observed that developed formulations were characterized by no phase separation. The suspended POS colored the hydrogel and cryogel base white. Properties of the POS-loaded formulations are presented in Table 1. The size of drug particles suspended in the hydrogel carrier is a key parameter influencing the effectiveness of the treatment. Smaller particles dissolve faster, which determines better absorption, but larger particles may be perceptible during application and may be felt as graininess by the patient during application. POS particles were uniformly distributed in the vehicle with mean sizes ranging from approximately 4.06 ± 1.82 μm to 4.99 ± 2.05 μm (Table 1).

**Figure 2:**
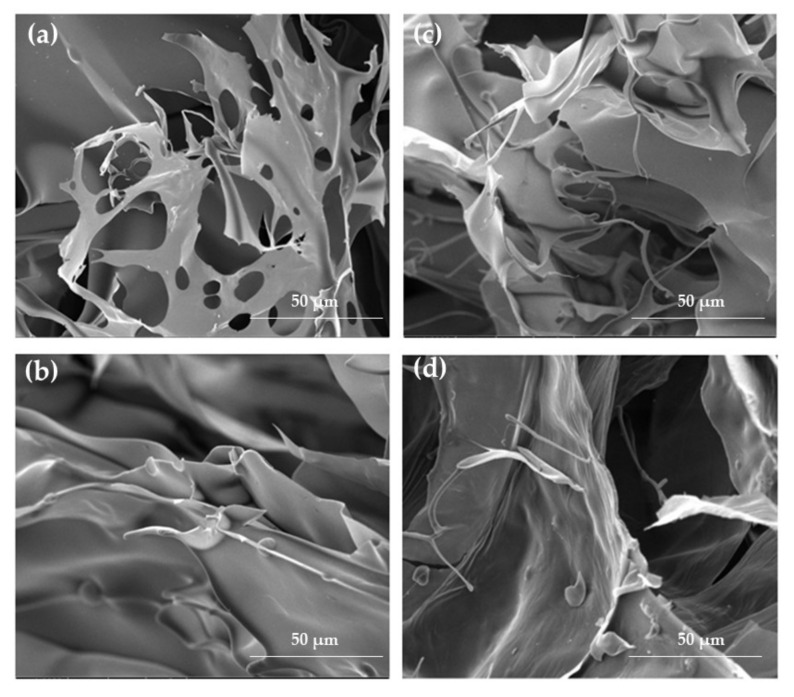
The representative SEM images of formulation (**a**) H0, (**b**) K0, (**c**) H2, and (**d**) K2 under magnification ×2000.

**Figure 3:**
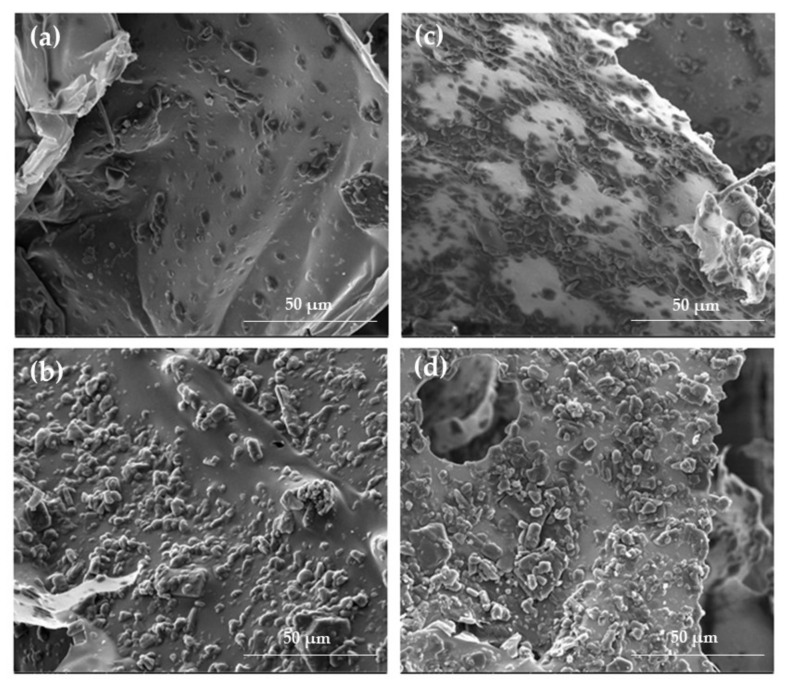
The representative SEM images of formulation (**a**) HP0, (**b**) KP0, (**c**) HP2, and (**d**) KP2 under magnification ×2000.

The SEM technique enabled observation of the morphology of hydro- and cryogels in both the placebo (Figure 2) and POS-loaded formulations (Figure 3). Depending on the composition, some differences in the formulations’ structure were observed. As shown in Figure 2a, formulation H0 expressed a porous structure. The samples of ALG/CS-G formulation (Figure 2c) seem to have an enhanced and more compact network compared to the ALG formulation. The freeze–thaw technique enabled to obtain cryogels characterized by compact and homogenous structure, both in the case of formulations composed of only ALG and ALG/CS-G (Figure 2b,d). In addition, the structure of POS-loaded cryogel formulations was more rough compared to traditional hydrogels (Figure 3).

The drug content analysis has shown that the average POS content was within the acceptable USP pharmacopoeial limit (90–110% of the labeled amount) [18], which might indicate that the drug particles were uniformly dispersed in all formulations (Table 1).

pH value is a crucial feature in the development of topical dosage forms, as it affects the solubility and stability of an active ingredient in the formulation. Although alkaline (pH 9–10) formulations applied to the skin might cause irritation, moderately acid pH (4.5–6.0) is the most beneficial for topical administration [19]. The pH values of prepared hydrogels were in the range from 4.93 ± 0.03 (H1) to 7.17 ± 0.02 (H0) and in the case of cryogels—from 5.00 ± 0.03 (K2) to 6.98 ± 0.04 (K0) (Table 1), which indicates that the pH range was suitable for dermal application. It was observed that insignificantly (*p* > 0.05) higher pH values were observed in the case of cryogels. pH values in both, hydrogel and cryogels formulations were only slightly higher after POS addition.

The viscosity affects the behavior of semi-solid formulations during application and contact time with the skin surface [2]. It was observed that the freeze–thaw technique significantly (*p* < 0.05) affected the viscosity in all formulations. Comparing hydrogel and cryogel formulations, even a twofold increase in viscosity was noticed, e.g., from 2986 ± 29 mPa∙s in formulation H0 to 4919 ± 89 mPa∙s in formulation K0 and from 3831 ± 942 mPa∙s in formulation H2 to 5644 ± 545 mPa∙s in formulation K2. The viscosity of both hydrogels and cryogels formulations was CS-G concentration-dependent—formulations with 2% CS-G were characterized by higher viscosity than formulations consisting only of ALG (H0 vs. H2 and K0 vs. K2) (Table 1). However, formulations H0.5 and HP0.5 composed of 2% ALG and 0.5% CS-G were characterized by lower viscosity than formulations with 1% and 2% CS-G due to an insufficient amount of CS-G to react with the ALG. Interestingly, both hydrogels and cryogels with 0.5% CS-G concentrations (H0.5 and K0.5) were characterized by lower viscosity than ALG formulations (H0 and K0), which proves the formation of weak bonds between polymers. In addition, the presence of POS in hydrogel and cryogel bases caused a decrease in their viscosity values (Table 1).

### 2.2. Bioadhesion

The skin, which is a protective barrier to the external environment, accounts for more than 10% of the body mass and is the largest organ of the body. In addition, the skin barrier prevents drug molecules topically applied from reaching deeper layers of the skin [20]. To exert the therapeutic effect, drug molecules must be released from the drug form and then overcome resistance in the course of skin permeation. Bioadhesive hydrogel formulations through adhesion to the skin, prolong the contact time of the formulation at the site of application, which improves penetration and the efficiency of the local therapy. In addition, through skin hydration, hydrogels improve drug permeation [21].

**Figure 4:**
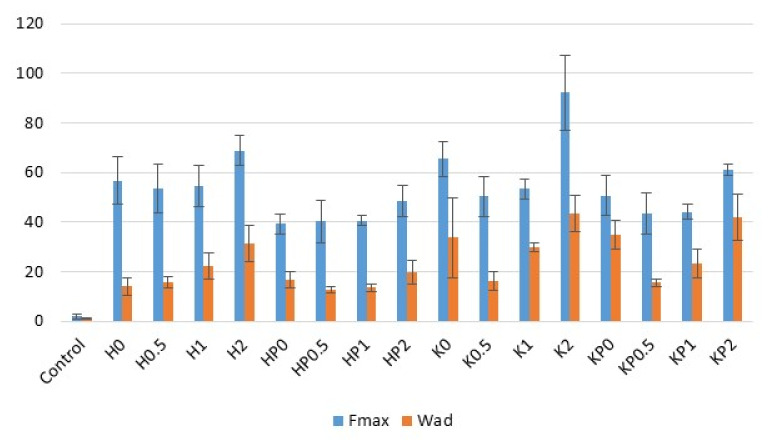
Bioadhesive properties (F_max_ and W_ad_) of hydrogels placebo (formulations H0–H2), POS-loaded hydrogels (formulations HP0–HP2), cryogels placebo (formulations K0–K2), and POS-loaded cryogels (formulations KP0–KP2) and cellulose disc (Control) (mean ± SD, *n* = 6).

ALG is an anionic polymer, which through its bioadhesive properties is widely utilized in the development of topical formulations and dressings [3]. CS is the cationic polymer and its amine groups interact through hydrogen bonding and ionic interactions with sialic acid, one of the components of mucin. However, at a pH above 6.0–6.5 CS loses its mucoadhesive properties, which is related with the decrease in charge density. Therefore CS derivatives with increased water solubility are increasingly used to develop mucoadhesive drug forms [22,23]. Moreover, it was reported that ALG-CS complexes possess greater adhesive properties than individual polymers [14,24]. To evaluate the behavior of designed hydrogel and cryogel formulations in contact with the skin surface, bioadhesive studies were performed. Hairless mice skin as the advantageous adhesive layer for the simulation of the in vivo conditions was utilized [25]. The effect of the freeze–thaw method, CS-G concentration and POS presence on the force of detachment (F_max_) and work required to overcome the hydrogel–skin interaction (W_ad_) is presented in Figure 4. It was observed that all examined hydrogels and cryogels showed beneficial adhesive properties. The obtained data have shown that cryogels were characterized by a significant (*p* < 0.05) increase in bioadhesiveness. Hydrogels placebo possessed F_max_ with the range from 53.60 ± 9.94 mN to 68.83 ± 6.05 mN, and W_ad_—from 14.04 ± 3.40 μJ to 31.25 ± 7.37 μJ. In the case of cryogels, F_max_ and W_ad_ were from 50.40 ± 7.99 mN to 92.25 ± 15.06 mN and from 16.20 ± 3.64 μJ to 43.32 ± 7.35 μJ, respectively. It was observed that the bioadhesive properties of hydrogels and cryogels were CS-G concentration-dependent. The lowest adhesive properties expressed formulations with 0.5% CS-G, while the highest—formulations with 2% CS-G. Similar results were reported by Chavda et al.—with the increasing amount of CS, the bioadhesion of the superporous hydrogel composite was increased [26]. As shown in Figure 4, POS presence led to a decrease in the F_max_ and W_ad_ values compared to the placebo formulations. This fact was also observed in the work of Wróblewska et al., where ALG-based hydrogels containing metronidazole were characterized by lower W_ad_ compared to placebo [27]. The highest bioadhesive properties, both in the hydrogels (HP2) and cryogels (KP2) were observed when 2% CS-G was applied.

### 2.3. Rheological and Textural Properties

**Figure 5:**
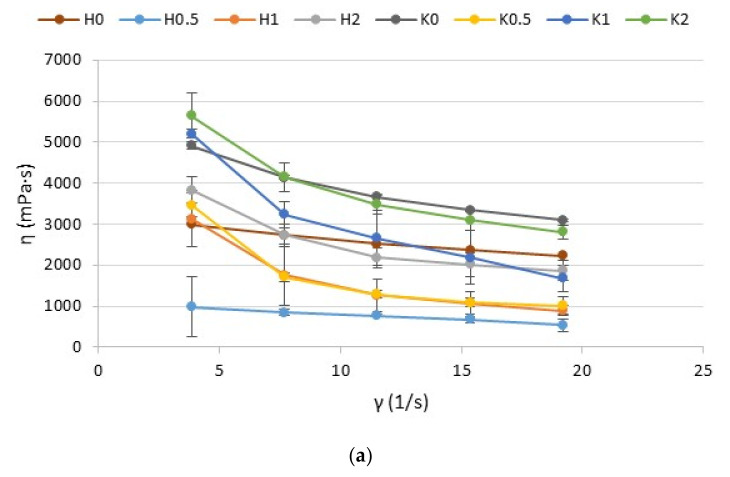

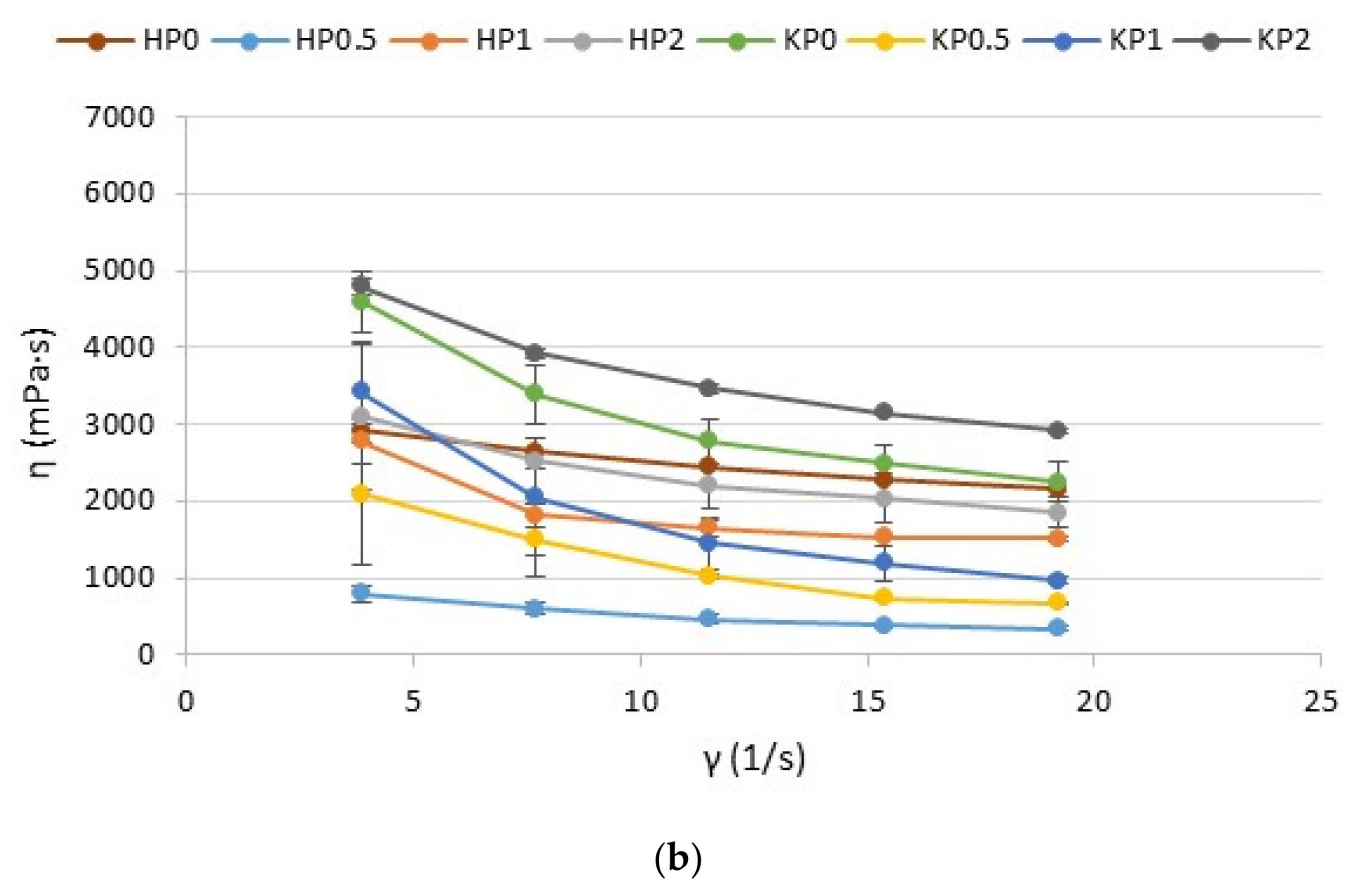
Plots of viscosity (η) vs. shear rate (γ) of the (**a**) hydrogels placebo formulations (H0–H2), cryogels placebo formulations (K0–K2), (**b**) POS-loaded hydrogels formulations (HP0–HP2), and POS-loaded cryogels formulations (KP0–KP2) (mean ± SD, *n* = 3) measured at 22 ± 1 °C.

Rheological properties are useful parameters in the evaluation of gel structure behavior. Rheological tests of all prepared formulations were expressed as a flow curve—a graph of the dependence of viscosity (η) on the shear rate (γ) (Figure 5).

The obtained results demonstrated that all investigated hydrogels and cryogels exhibited shear-thinning behavior, which is characterized by a reduction in the viscosity with an increase in the shear stress gradient and indicates their non-Newtonian nature. This phenomenon is typical to polymer solutions and expresses the character of a pseudo-plastic fluid where the viscosity is decreased under the influence of shear [28]. The shear-thinning phenomenon is an important parameter for topical application—it enables the thin gel layer formation on the skin during application [29]. The viscosity reduction, under the shear force, is associated with the destruction of the gel structure. Similar results were observed by Petrova et al. in the ALG/CS hydrogel with tetracycline hydrochloride—when CS concentration in the ALG/CS hydrogel was increased—the increase of the yield stress, maximum Newtonian viscosity, and relaxation time were observed [30].

The texture analysis is a crucial test during the optimization of topical formulations as it provides parameters such as firmness, compressibility, adhesiveness and cohesiveness. These factors supply information about the response of preparations in the contact with external force during removal from the container, about the spreadability and retention time at the application site. Dosage forms for skin application should be characterized by suitable mechanical properties enabling easy removal from the container and spreading over the surface, and by adequate firmness and compressibility. Firmness is defined as the maximal force required to attain a given deformation and it characterizes the applicability of the gel to the desired site. The preparation is easily distributed when firmness reaches relatively low values. Compressibility characterizes gel deformation under compression and it is expressed as the work required to deform the product during compression of the probe. Low compressibility means easy removal of the formulation from the container and enables high spreadability at the application site. Cohesiveness determines the effect of repeated shearing stress on the structural properties of the analyzed gels. The high value of cohesiveness enables full structural recovery during gel application [31,32,33]. Finally, adhesiveness, which is closely related to bioadhesion, represents the work required to remove the sample, which is connected with the breaking of cohesive bonds. Therefore, higher values are desirable to ensure prolonged adhesion.

**Figure 6:**
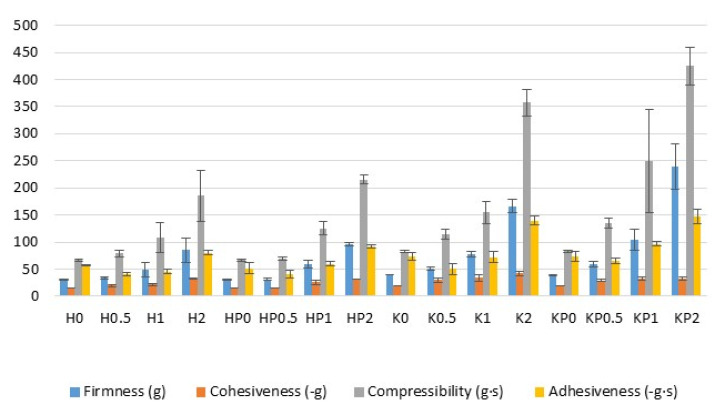
Textural properties of hydrogels placebo (formulations H0–H2), POS-loaded hydrogels (formulations HP0–HP2), cryogels placebo (formulations K0–K2) and POS-loaded cryogels (formulations KP0–KP2) (mean ± SD, *n* = 3).

Based on the results of the texture analysis, it was shown that all formulations were characterized by suitable mechanical properties for skin application (Figure 6). Cryogels were characterized by significantly (*p* < 0.05) higher values of firmness, compressibility, cohesiveness and adhesiveness compared to traditional hydrogels. The highest values of firmness, adhesiveness and compressibility were reported for obtained formulation KP2. It was observed that the placebo formulation possessed slightly lower firmness, compressibility and adhesiveness compared to POS-loaded hydro- and cryogels. This fact is compatible with the research conducted by Rençber et al., who observed that the presence of nystatin in ALG/CS hydrogel affected the increase in rheological properties of the formulations [34]. On the other hand, Sezer et al. reported an increase in gel adhesiveness with the increasing CS concentrations from 1.5% to 2%, but the cohesiveness was found to be negatively correlated [35]. Cryogel formulations were characterized by significantly higher values of firmness, compressibility and adhesiveness compared with hydrogel formulations (Figure 6). The POS addition caused an increase in values of firmness, compressibility, cohesiveness and adhesiveness. It was observed that the mechanical parameters were CS-G concentration-dependent and the highest values were recorded in the formulations with 2% CS-G. The obtained results suggest that the freeze–thaw technique and CS-G addition method significantly improved the mechanical properties of ALG gels.

### 2.4. In Vitro POS Release 

**Figure 7:**
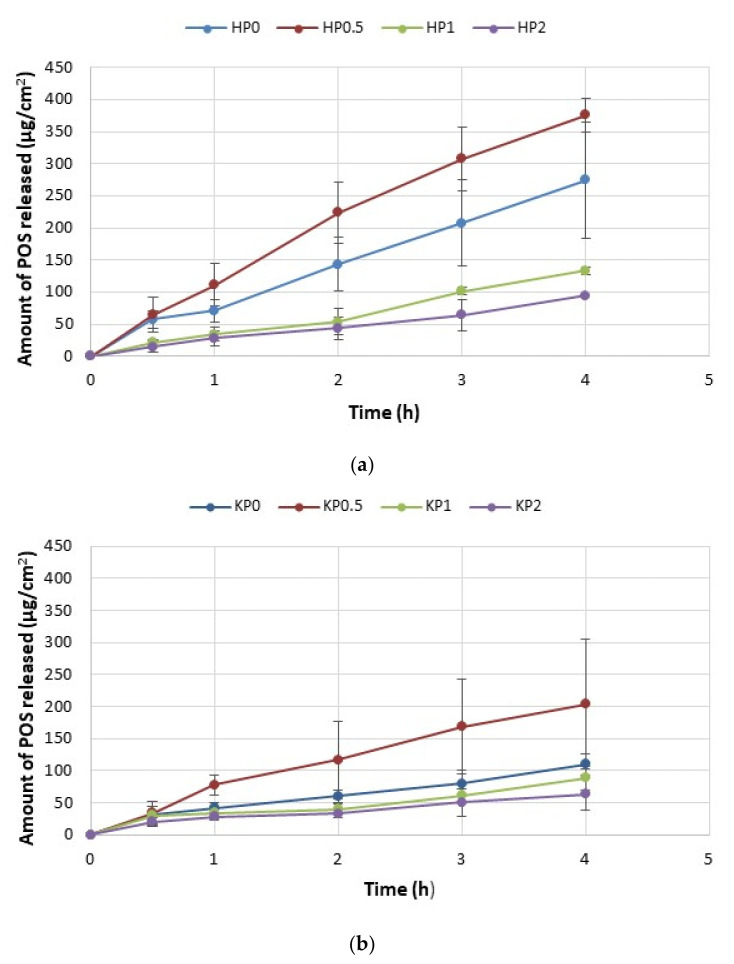
POS release from (**a**) hydrogels formulations HP0–HP2 and (**b**) cryogels formulations KP0–KP2 (mean ± SD, *n* = 3).

The drug release from topical dosage formulations is a complex process influenced by many factors such as viscosity, pH and manufacturing process, the composition of the vehicle or physicochemical characteristics of formulation ingredients [36]. The release process has a meaningful impact on drug action at the skin surface or its deeper layers. The release of POS from all formulations is presented in Figure 7. It was shown that the freeze–thaw technique significantly affected the release profile—POS release from ALG/CS-G cryogels was significantly (*p* < 0.05) sustained in comparison to traditional hydrogel formulations. POS cumulative amount released after 4 h from hydrogels was from 93.98 ± 24.86 μg/cm^2^ (in HP2) to 274.38 ± 90.02 μg/cm^2^ (in HP0.5) (Figure 7a). The most sustained POS release was observed for the formulation KP2 (after 4 h, the cumulative amount of released POS was 63.69 ± 24.86 μg/cm^2^) (Figure 7b). This fact is closely related to the viscosity of the formulations. Cryogel formulations were characterized by higher values of viscosity and, as a consequence, by prolonged POS release. After 4 h from formulation HP0.5 (with the lowest viscosity of 794 ± 108 mPa∙s) 375.43 ± 25.94 μg/cm^2^ POS was released, but from the formulation with the highest viscosity (KP2—4798 ± 108 mPa∙s)—the amount of released POS was only 63.69 ± 24.86 μg/cm^2^. Additionally, it was observed that sustained POS release was CS-G concentration-dependent—when CS-G concentration was increased, the amount of released POS was decreased. It is in the agreement with the results obtained by Bhutani et al. They found that the increase in ALG hydrogel viscosity affected piperine release [37].

The mechanism of POS release from prepared formulations was examined according to different mathematical kinetic models (Table 2). Interestingly, the obtained data with zero-order as well as with first-order kinetics in all formulations and correlation coefficients were from 0.970 for KP2 to 0.995 for KP0. The obtained data indicated that POS release plots for formulations HP0–HP2 showed maximum linearity with high regression values (R^2^) in the Higuchi model and in the Korsmeyer–Peppas equation, suggesting that the release mechanism was diffusion-based. The mechanism of the in vitro drug release is well explained by the Korsmeyer–Peppas model, where diffusion exponent *n* is an important parameter describing the mechanism of the drug release [38]. It was observed that in all formulations diffusion exponent *n* possessed values from 0.293 to 0.652. The obtained results were shown that *n* values in cryogels formulations were from 0.293 in formulation KP1 to 0.425 in formulation KP0.5, which indicated POS release according to the Fickian diffusion. Hydrogel formulations were characterized by higher *n* values (from 0.422 for HP0 to 0.652 for HP1). This fact indicates that POS release from hydrogel matrices was according to the anomalous transport. In the case of gel drug delivery systems, when *n* > 0.45—the release mechanism follows a non-Fickian transport. This anomalous (or non-Fickian) mechanism is known in the literature as pseudo-Fickian diffusion, indicating that in the first step of drug release this process is limited by swelling [38]. It is consistent with the results obtained by Nikolova et al., who observed *n* values greater than 0.45 for the release of diclofenac sodium from CS/ALG hydrogels composed of 0.05% (*w/w*) CS and 0.05% (*w/w*) ALG solutions in ratio 1:2 [39].

In the development of drug dosage forms it is crucial to explain the drug release mechanism, but also to evaluate if the utilization of a new technological process affects the drug release. Therefore, the difference index (*f*_1_) and similarity index (*f*_2_) [40] were utilized to determine the POS similarity dissolution profiles. According to these factors, when values of *f*_1_ are in the range 0–15 and *f*_2_ in the range 50–100, the release profiles are comparable [41]. In order to take into account the influence of the freeze–thaw process on the drug release, the hydrogels and cryogels formulations were compared (HP0.5 vs. KP0.5, HP1 vs. KP1 and HP2 vs. KP2). It was observed that all collated formulations had a similarity factor *f*_2_ < 50 and difference factor *f*_1_ > 15, which suggests that the freeze–thaw technique significantly affected the POS release profile.

### 2.5. DSC Analysis

**Figure 8:**
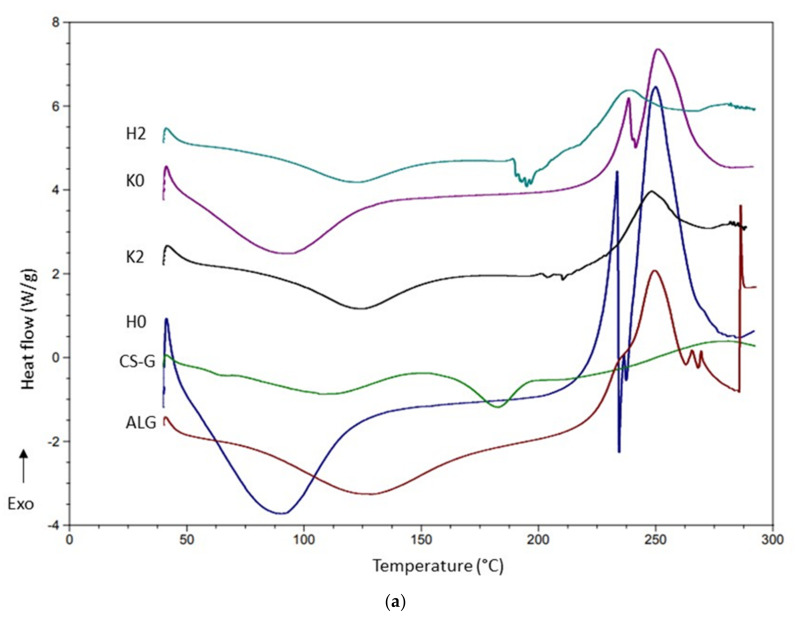

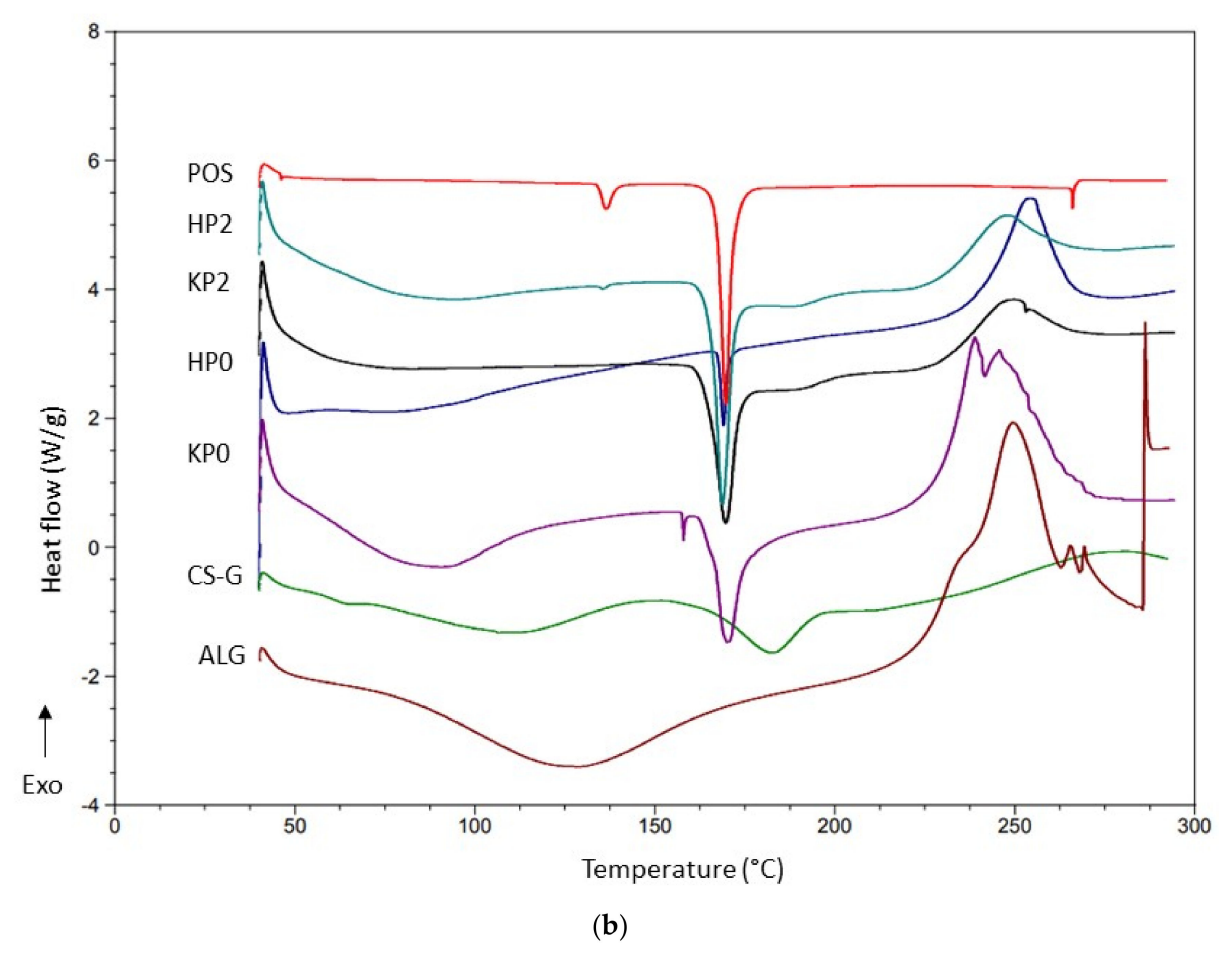
DSC thermograms of (**a**) sodium alginate (ALG), chitosan glutamate (CS-G), formulations placebo (H0, K0, H2, K2) and (**b**) sodium alginate (ALG), chitosan glutamate (CS-G), posaconazole (POS) and POS-loaded formulations (HP0, KP0, HP2, KP2).

DSC is a method utilized in pharmaceutical analysis for obtaining detailed information regarding the physical and energetic characteristics of active substances and excipients, the occurrence of potential interactions and the presence of possible impurities [42]. Thermograms of the hydrogels placebo (formulation H0, H2), POS-loaded hydrogels (formulation HP0, HP2), cryogels placebo (formulation K0, K2), POS-loaded cryogels (formulation KP0, KP2) and their components are shown in Figure 8.

ALG thermograms expressed broad endothermal peaks between 100 °C and 150 °C which correlates with water evaporation related to hydrophilic groups [24,43,44]. Additionally, a sharp exothermic peak related to polymer decomposition at 248 °C was observed. In the case of formulations H0 and K0, a shift of endothermic peaks towards lower temperatures was observed. Additionally, peaks with complicated shapes (exothermic peaks at 233 °C and 249 °C in formulation H0 and at 238 °C and 250 °C in K0) overlapping with another endothermic process at 238 °C (H0) and at 240°C (K0) were observed (Figure 8a) [45,46]. Dudek et al. suggest that ALG exothermic peaks are the result of the polymer backbone degradation in the dehydration process, depolymerization and saccharide ring destruction which might be caused by partial decarboxylation of the protonated carboxylic groups [45]. The thermogram of pure CS-G showed two endothermic peaks—a broad peak between 50 °C and 100 °C caused by water evaporation and sharp—at 182.98 °C, which might suggest glass transition [47]. The thermogram of POS exhibited two sharp endothermic peaks, at temperatures 138.73 °C indicating the loss of crystal water, and at 172.70 °C suggesting the melting point of pure POS [43]. In thermograms, no temperature shifts of POS were observed which suggests no interaction between drug and polymer for all systems (Figure 8b). The shape changes of broad endothermal peaks in ALG/CS-G formulations compared to unprocessed ALG and CS-G might be related to interaction between both polymers [46,48]. In addition, thermograms of the H2, HP2 and K2, KP2 formulations did not express peaks of individual ALG and CS-G, which proved the interactions between ALG and CS-G (Figure 8a,b) [48,49].

### 2.6. Antifungal Activity

**Figure 9:**
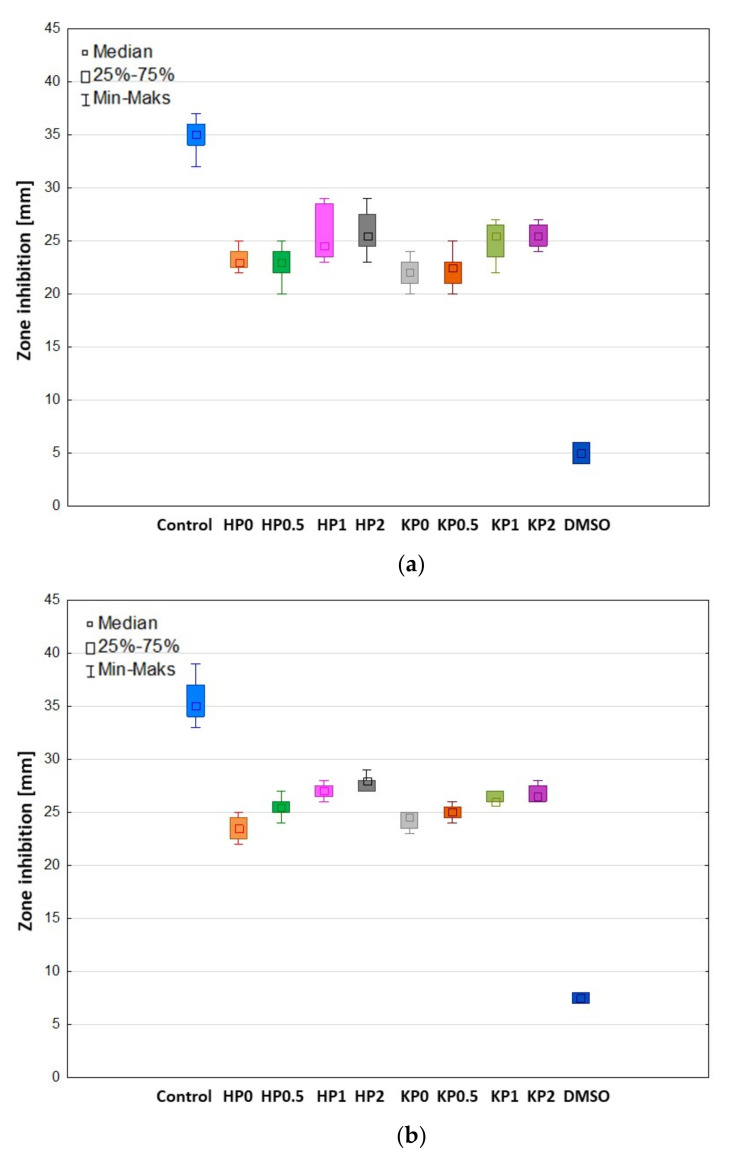

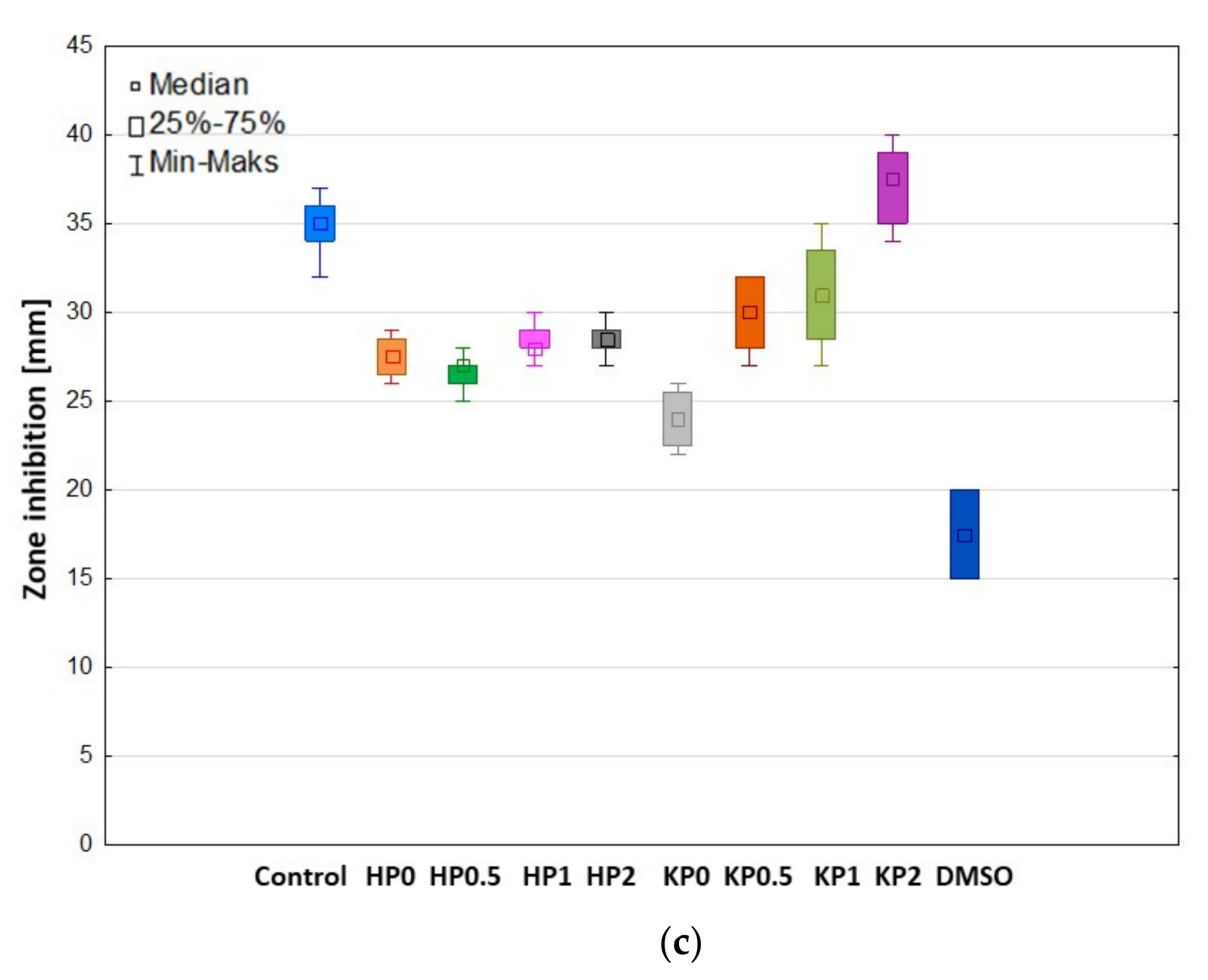
Box-plot graphs presenting antifungal activity of POS-loaded hydrogels (formulations HP0–HP2) and POS-loaded cryogels (formulations KP0–KP2); POS/DMSO (Control) against (**a**) *C. albicans*, (**b**) *C. krusei* and (**c**) *C. parapsilosis* (*n* = 3).

**Figure 10:**
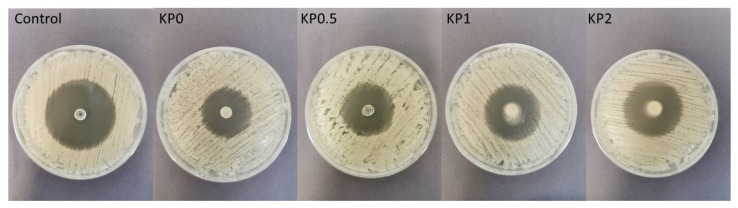
Representative images of antifungal activity of cryogel formulations KP0–KP2 and POS/DMSO (control) against *C. parapsilosis*.

**Figure 11:**
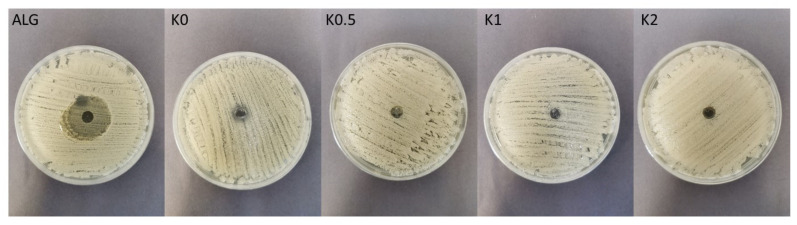
Representative images of antifungal activity of ALG in a powder form and cryogel formulations (K0–K2) against *C. parapsilosis*.

The antimicrobial properties of natural polymers have been known for a long time. Despite wide ALG utilization in drug formulation design, there are only a few papers indicating its antifungal activity per se [50]. The mechanism of ALG antifungal action is still not explained, however, there are hypotheses indicating that ALG can form a layer on the microbial cell and thus disturb the nutrients transport, which imbalances the function of the cell membrane [51]. Moreover, ALG is negatively charged, so it can interact with the surface of the microbial cell providing the leak of the intracellular content [52]. In contrast to ALG, many studies of the antifungal activity of CS have been carried out and the mechanism of its action is well documented. The protonated amino groups of CS with positive charge interact with negatively charged phospholipid components of fungi membrane, which leads to an increase in the membrane permeability and the leakage of intracellular substances (e.g., phosphate, nucleotides, and K^+^ ions). Therefore, as a result of these processes, the transport of nutrients is disturbed, which leads to the loss of metabolic capabilities, e.g., respiration and fermentation, and in effect—to the inhibition of fungi growth [53]. The antifungal activity of POS-loaded hydrogels and cryogels on *Candida* spp. was measured after 24 h and 48 h of incubation (Figure 9, Figure 10 and Figure 11). In our previous study, it was shown that films placebo composed of only high molecular weight ALG and low molecular weight oligosaccharides possessed antifungal activity [54]. Thus, it was surprising that hydrogels and cryogels placebos did not have the ability to inhibit the growth of all tested *Candida* strains (Figure 11). This fact might be explained by the lower concentration of polymers in gel formulations. Figure 11 shows ALG antifungal activity in the form of a powder against the strain of *C. parapsilosis.* POS-loaded hydrogel formulations were characterized by slightly higher inhibition of *C. albicans* and *C. krusei* growth than cryogel formulations (Figure 9a,b). This fact might be correlated with their viscosity—hydrogels possessed lower values of viscosity than cryogels which probably affected their better penetration through the agar medium. Interestingly, it was shown that ALG/CS-G cryogel formulations exhibited higher inhibition zone values in *C. parapsilosis* culture which ranged from 26.0 ± 1.6 mm for KP0.5 to 28.7 ± 3.9 mm for KP2 (Figure 9c). Additionally, it was observed that CS-G presence improved antifungal activity of tested formulations, which was especially expressed in *C. parapsilosis* culture. Similar observations were found by Peña et al., who examined the effect of low molecular weight (96.5 KDa) CS in different concentrations on the growth of *C. albicans*. They have shown that in the case of higher CS concentrations, the growth of *C. albicans* was stronger inhibited [55].

## 3. Materials and Methods

Posaconazole (POS) was provided by Kerui Biotechnology Co. LTD (Xi’an, China). Sodium alginate (ALG) was obtained from *Macrocystis pyrifera*, with medium viscosity (2415 mPa∙s for 1% solution at 25 °C, 61% mannuronic acid (M) and 39% guluronic acid (G), molecular weight 3.5 × 10^5^ Da, M/G ratio of 1.56) was purchased from Sigma Aldrich St. Louis, MO, USA. Chitosan glutamate, degree of deacetylation 80–95 %, viscosity of 1% solution in water at 25° C 21.4 mPas and MW 30–600 kDa was purchased from Heppe Medical CS GmbH (Haale, Germany). Water was distilled and passed through a reverse osmosis system Milli-Q Reagent Water System (Billerica, MA, USA). Sodium chloride, potassium phosphate monobasic, sodium phosphate dibasic and sodium dodecyl sulfate (SDS) were from Chempur (Piekary Śląskie, Poland). Ethanol 99.8% was obtained from Avantor Performance Materials Poland S.A. Methanol and acetonitrile were of HPLC grade and were purchased from Merck (Darmstadt, Germany). All other ingredients used were of analytical grade. Natural cellulose membrane—Cuprophan^®^ (molecular weight cut-off 10,000 Da) was purchased from Medicell (London, UK). Hairless mouse skin was obtained from the Experimental Medicine Center of the Medical University of Białystok. The skin was collected from the dorsal area of Cby.Cg-Foxn1nu/cmdb hairless mice intended for the collection of organs. This procedure did not require the approval of the Local Ethical Committee for Experiments on Animals. Samples of the skin were frozen at −20 °C prior to testing and stored up to a maximum of one month. After defrosting, the samples were split into 5 mm diameter fragments. All other ingredients used were of analytical grade. The stock cultures of *C. albicans*, *C. parapsilosis*, and *C. krusei* from American Type Culture Collection (ATCC), and Sabouraud dextrose agar were provided by Biomaxima (Lublin, Poland).

### 3.1. Preparation of Hydrogels

In the first step, a 2% ALG solution (based on the preliminary studies) was prepared by dissolving an appropriate amount of polymer in purified water and stirred using an RZR 2020 mechanical stirrer (Heidolph Instruments, Schabach, Germany) to obtain homogenous dispersion. Then, different amounts of CS-G were dissolved in purified water in order to receive 0.5%, 1% and 2% CS-G concentrations (Table 3). In the next step, polymer solutions were adjusted to pH 5.0 to provide interaction between CS-G and ALG [24]. Then, CS-G solutions were injected dropwise into the ALG solution under constant stirring to obtain homogenous hydrogels [56]. POS at 2.0% *w/w* concentration was uniformly dispersed in gel vehicles. Control gel formulations without POS (placebo) were also initially prepared.

### 3.2. Preparation of Cryogels by Freeze–Thaw Method

To obtain cryogels, hydrogel formulations were frozen at −20 °C for 18 h followed by thawing at room temperature for 6 h [54]. The freeze–thaw cycle was carried out three times. POS-loaded cryogels were obtained by the dispersion of active substance (2.0%, *w/w*) in prepared cryogels (Table 3).

### 3.3. pH Determination

The pH of obtained hydrogels and cryogels was determined after 24 h of preparation by using a glass electrode of the pH-meter Orion 3 Star (ThermoScientific, Waltham, MA, USA).

### 3.4. Scanning Electron Microscope (SEM) Analysis

For SEM analysis, 5 g of the representative hydrogels H0, H2, HP0, HP2 and cryogels K0, K2, KP0, KP2 was placed in glass vials and the lyophilization process was carried out at −20 °C for 24 h by using freeze-dryer Christ Alpha 1–2 LDplus (Martin Christ, Osterode am Harz, Germany) equipped with a rotary vane pump RZ 2.5 (Vacuubrand, Wertheim, Germany). The lyophilizates cross-sections were evaluated with scanning electron microscopy (Inspect™S50, FEI Company, Hillsboro, OR, USA). The samples were coated with a 6 nm gold layer to improve the conductivity of the electrons. The microscope worked in the high vacuum mode. The SED (secondary electron detector) and the voltage of 10 kV (due to the sample decomposition) were used. The working distance from the detector was 10 mm. The images were taken at magnifications of 100×, 150×, 1000×, 2000×, 5000× and 10,000×.

### 3.5. Particles Size Analysis

Hydrogels and cryogels were observed using an optical microscope Motic BA 400 (Moticon, Wetzlar, Germany) using total magnification 100×, 400× and 1000×. For analysis, 1 mg of each hydrogel and cryogel formulation was placed on a glass slide. POS particles were observed and measured in at least three different areas of observation [27].

### 3.6. Drug Content 

POS content was determined after extraction of 1 g hydrogel or cryogel formulation samples in 10 mL of ethanol (99.8%). The samples were assayed at 260 nm using Genesys 10S UV–vis spectrophotometer (Thermo Scientific, Madison, WI, USA). The standard calibration curve was linear over the range of 10–100 µg/mL (R^2^ = 0.999).

### 3.7. Ex Vivo Bioadhesiveness

Bioadhesiveness was tested by using TA.XT.Plus Texture Analyzer (Stable Micro Systems, Godalming, UK) with A/MUC equipment and hairless mice skin to imitate the in vivo conditions. While analysis, pieces of skin or cellulose discs (5 mm in diameter) were adhered by cyanoacrylate glue on the upper probe whilst the hydrogel sample (1.0 mL) was set on the platform below the texture analyzer probe. The temperature was maintained at 32  ±  1 °C. Process parameters, chosen during preliminary tests, were as follows: pretest speed 0.5 mm/s, test speed 0.1 m/s, contact time 180 s, post-test speed 0.1 mm/s, applied force 1 N. Bioadhesiveness was expressed as the detachment force (F_max_) and the work of adhesion (W_ad_). The work of adhesion (W_ad_) was calculated using the following formula:

W_ad_ = A × 0.1 × 1000 (1)

where A—area under the force versus distance curve, multiplication by 0.1—conversion time measurement to distance (the sampler was raised at 0.1 mm·s^−1^), then multiply by 1000 in order to express the result in units of work, µJ [27].

### 3.8. Viscosity and Rheological Properties Analysis 

The study of the rheological properties of all formulations was performed by using Brookfield rotational viscometer (RVDV-III Ultra, Brookfield Engineering Laboratories, Middleboro, MA, USA) with a CPA52Z cone (plate diameter 24 mm, cone angle 3°) measuring system, at a temperature of 22 ± 1 °C. The viscosity values of hydrogel and cryogel formulations (0.5 g) at a shear rate of 10.00 s^−1^ were recorded. The rheological properties were presented as rheogram dependences of viscosity (η) on the shear rate (γ) (2.00–20.00 s^−1^).

### 3.9. Texture Analysis

Texture analysis was evaluated by using a TA.XT Plus texture analyzer (Stable Micro System, Godalming, UK) for backward extrusion measurements. A disc (35 mm diameter) was pressed at a speed of 2 mm·s^−1^ for a distance of 5 mm into the sample (30 g) and redrawn. Data collection and analysis were performed using the Texture Exponent software. Parameters such as firmness, cohesiveness, compressibility, and adhesiveness were determined based on the force–time plots.

### 3.10. In Vitro POS Release

The in vitro POS release was examined through natural cellulose membrane using an enhancer cell with a surface area of 3.80 cm^2^. The enhancer cell consisted of a Teflon load ring, a cap, a membrane, and a drug reservoir. This study was carried out using the USP dissolution apparatus 2 (Agilent 708-DS, Agilent Technologies, Cary, NC, USA) with mini vessels (250 mL) and mini paddles. Each hydrogel sample weighed about 2 g was placed in the enhancer cell, which was then immersed in the dissolution vessel containing 150 mL of the release medium (phosphate buffer pH 6.0 with the addition of 1% SDS to obtain the sink conditions), previously warmed and maintained at 32 ± 1 °C (adequate to the skin surface temperature). Agitation was performed by mini paddles at 75 rpm and aliquots each of 1 mL were withdrawn at different time intervals (0.5, 1, 2, 3 and 4 h). Withdrawn samples were replaced by equal volumes of fresh release medium. The samples were assayed at 240 nm using high-performance liquid chromatography (HPLC) described in point 3.11.

### 3.11. High-Performance Liquid Chromatography (HPLC) Assay

POS concentration was determined by the HPLC method using an Agilent Technologies 1200 system (Agilent, Waldbronn, Germany) equipped with a Poroshell^®^ 120 EC-C18 2.7 μM ODS 4.6 × 150 mm, 2.7 μm column (Agilent, Waldbronn, Germany). For the mobile phase, acetonitrile: methanol: water (60:20:20, *v*/*v*) with a flow rate of 0.5 mL/min was applied [54]. The UV detection was operated with a wavelength of 240 nm. The retention time of POS was observed at 5.3 min. The standard calibration curve was linear over the range of 1–20 μg/mL with a correlation coefficient (R^2^) of 0.999.

### 3.12. Mathematical Modeling of POS Release Profile

To expound the mechanism of POS release, obtained data were analyzed according to zero-order kinetics, first-order kinetics, the Higuchi model and the Korsmeyer–Peppas equation. The constants of release kinetics and the regression coefficients (R^2^) were evaluated from the slope of plots by linear regression analysis. For the Korsmeyer–Peppas model, the fraction of drug remaining at time t was determined for every time interval log (Mt/M∞) and plotted against the log of time t. The slope of the line was taken as the value of *n*—diffusional release exponent used for the interpretation of release mechanism [57,58].

### 3.13. Index of Similarity and Dissimilarity

To compare release profiles of designed hydrogels and cryogels, a model utilizing a difference factor *f*_1_ and similarity factor *f*_2_ was applied. The difference index *f*_1_ was calculated by the formula:

*f*_1_ = {(∑ = 1n|R_t_ − T_t_|) (∑ = 1nR_t_)} × 100 (2)

The similarity index *f*_2_ indicates the potential release similarity and it is calculated by the following equation:

*f*_2_ = 50 × Log {[1 + (1/*n*)∑ = 1*n*(R_t_ − T_t_) × 2] − 0.5 × 100} (3)

where *n*—number of samples, R_t_ and T_t_—data of drug dissolution of control and test sample at the same time (t). The values of *f*_1_ in the range 0–15 and *f*_2_ between 50–100 indicate the release profiles’ similarity between samples [40,41].

### 3.14. Differential Scanning Calorimetry (DSC) 

DSC analysis was accomplished by a thermal analyzer system (DSC TEQ2000, TA Instruments, New Castle, DE, USA). Samples (in the amount of 2 mg) of placebo H0, K0, H2, K2 and POS-loaded formulations—HP0, KP0, HP2, KP2 and unprocessed ALG, CS-G, and POS were placed in aluminum pans and heated in the range from 25 °C to 300 °C at a scanning rate of 10 °C/min under argon flow of 20 mL/min. An empty pan sealed was used as a reference.

### 3.15. Antifungal Activity

To evaluate the antifungal activity of designed formulations, the agar diffusion method was used. Petri dishes containing Sabouraud’s dextrose agar were seeded with 50 µL of the fungal inoculums prepared using sterile 0.9% NaCl solution, with a final density of 5 × 10^6^ CFU/mL (corresponding to 0.5 in the McFarland scale) [59]. The optical density of inoculum was measured spectrophotometrically at 550 nm using a Genesys 10S UV–vis spectrophotometer (Thermo Scientific, Madison, WI, USA). After 15 min of drying at room temperature, wells with a diameter of 5 mm were cut out in agar plates, into which prepared gel formulations (100 mg) were placed. As controls, 50 µL of a solution obtained by dissolving POS in DMSO (corresponding to 10 mg of POS) and formulations placebo were used. Plates were incubated at 37 ± 0.1 °C for 24 and 48 h. After this time the growth inhibition zones were measured using a caliper (Mitutoyo, Kawasaki, Japan) with an accuracy of 0.1 mm.

### 3.16. Statistical Analysis

The obtained data were performed using Statistica 12.0 software (StatSoft, Tulsa, OK, USA). Quantity variables were expressed as the mean and standard deviation. One-way analysis of variance (ANOVA), Kruskal–Wallis test was used as statistical tests. Differences between groups were examined to be significant at *p* < 0.05.

## 4. Conclusions

In this study, hydrogels and cryogels based on ALG and CS-G with POS as a model antifungal substance were prepared. The freeze–thaw technique significantly improved viscosity, bioadhesiveness and textural properties. In addition, in vitro POS release from cryogels was prolonged, according to the Fickian diffusion. Moreover, it was observed that ALG/CS-G cryogel formulations exhibited higher values of inhibition zone of *C. parapsilosis* than hydrogel formulations. The obtained results might be a basis for further research on the application of ALG/CS-G cryogels obtained by the freeze–thaw technique as topical drug carriers for antifungal drugs.

## Figures and Tables

**Table 1:** Characteristics of designed hydrogels placebo (formulations H0–H2), POS-loaded hydrogels (formulations HP0–HP2), cryogels placebo (formulations K0–K2), and POS-loaded cryogels (formulations KP0–KP2) (mean ± SD, *n* = 3).

Formulation	pH	Mean Particle Size (μm)	Drug Content (%)	Viscosity * (mPa∙s)
H0	7.17 ± 0.02	–	–	2986 ± 29
H0.5	5.05 ± 0.06	–	–	984 ± 73
H1	4.93 ± 0.03	–	–	3135 ± 675
H2	4.94 ± 0.02	–	–	3831 ± 942
HP0	6.78 ± 0.02	4.93 ± 2.45	100.93 ± 2.97	2916 ± 28
HP0.5	5.06 ± 0.01	4.75 ± 1.95	105.54 ± 2.10	794 ± 108
HP1	5.02 ± 0.03	4.99 ± 2.05	103.89 ± 1.20	2784 ± 290
HP2	5.04 ± 0.04	4.61 ± 2.73	98.11 ± 2.56	3107 ± 315
K0	6.98 ± 0.04	–	–	4919 ± 89
K0.5	5.14 ± 0.04	–	–	3469 ± 256
K1	5.12 ± 0.01	–	–	5204 ± 110
K2	5.00 ± 0.03	–	–	5644 ± 545
KP0	6.80 ± 0.05	4.45 ± 2.09	100.58 ± 3.07	4591 ± 392
KP0.5	5.18 ± 0.02	4.28 ± 1.45	97.69 ± 8.45	2097 ± 915
KP1	5.15 ± 0.01	4.60 ± 1.71	100.65 ± 4.06	3418 ± 659
KP2	5.07 ± 0.02	4.06 ± 1.82	98.29 ± 2.36	4798 ± 108

* viscosity was measured at the shear rate 10.00 s^−1^ at 22 ± 1 °C.

**Table 2:** Models of POS release from hydrogels formulations HP0–HP2 and cryogels formulations KP0–KP2.

Formulation	Zero Order Kinetics		First Order Kinetics		Higuchi Model		Korsmeyer–Peppas Model		
	R^2^	K	R^2^	K	R^2^	K	R^2^	K	*n*
HP0	0.995	0.608	0.968	0.466	0.966	1.631	0.988	0.555	0.422
HP0.5	0.991	0.860	0.915	0.492	0.995	2.347	0.973	0.600	0.499
HP1	0.980	0.308	0.975	0.519	0.937	0.824	0.992	0.629	0.652
HP2	0.987	0.205	0.955	0.488	0.951	0.549	0.988	0.587	0.645
KP0	0.988	0.207	0.955	0.488	0.952	0.554	0.997	0.414	0.341
KP0.5	0.984	0.448	0.859	0.466	0.994	1.229	0.937	0.573	0.425
KP1	0.975	0.155	0.927	0.309	0.849	0.405	0.923	0.356	0.293
KP2	0.970	0.115	0.971	0.313	0.929	0.306	0.969	0.369	0.358

R^2^: correlation coefficient, K: release constant, and *n*: the release exponent.

**Table 3:** Composition of designed hydrogel placebo formulations (H0–H2), POS-loaded hydrogel formulations (HP0–HP2), cryogel placebo formulations (K0–K2) and POS-loaded cryogel formulations (KP0–KP2).

Formulation	ALG (%)	CS-G (%)	POS (%)
H0	2	–	–
H0.5	2	0.5	–
H1	2	1	–
H2	2	2	–
HP0	2	–	2
HP0.5	2	0.5	2
HP1	2	1	2
HP2	2	2	2
K0	2	–	–
K0.5	2	0.5	–
K1	2	1	–
K2	2	2	–
KP0	2	–	2
KP0.5	2	0.5	2
KP1	2	1	2
KP2	2	2	2

## Data Availability

Data are contained within the article.

## References

[1] Larrañeta E., Stewart S., Ervine M., Al-Kasasbeh R., Donnelly R.F. (2018). Hydrogels for hydrophobic drug delivery. Classification, synthesis and applications. J. Funct. Biomater..

[2] Chai Q., Jiao Y., Yu X. (2017). Hydrogels for biomedical applications: Their characteristics and the mechanisms behind them. Gels.

[3] Szekalska M., Puciłowska A., Szymańska E., Ciosek P., Winnicka K. (2016). Alginate: Current use and future perspectives in pharmaceutical and biomedical applications. Int. J. Polym. Sci..

[4] Kou S.G., Peters L.M., Mucalo M.R. (2021). Chitosan: A review of sources and preparation methods. Int. J. Biol. Macromol..

[5] Rinaldi F., Hanieh P.N., Chan L.K.N., Angeloni L., Passeri D., Rossi M., Wang J.T., Imbriano A., Carafa M., Marianecci C. (2018). Chitosan glutamate-coated niosomes: A proposal for nose-to-brain delivery. Pharmaceutics.

[6] Ardean C., Davidescu C.M., Nemeş N.S., Negrea A., Ciopec M., Duteanu N., Negrea P., Duda-Seiman D., Musta V. (2021). Factors influencing the antibacterial activity of chitosan and chitosan modified by functionalization. Int. J. Mol. Sci..

[7] Zhao D., Yu S., Sun B., Gao S., Guo S., Zhao K. (2018). Biomedical applications of chitosan and its derivative nanoparticles. Polymers.

[8] Taudorf E.H., Jemec G.B.E., Hay R.J., Saunte D.M.L. (2019). Cutaneous candidiasis-an evidence-based review of topical and systemic treatments to inform clinical practice. J. Eur. Acad. Dermatol. Venereol..

[9] Armstrong A.W., Bukhalo M., Blauvelt A. (2016). A clinician’s guide to the diagnosis and treatment of candidiasis in patients with psoriasis. Am. J. Clin. Dermatol..

[10] Wong T.Y., Loo Y.S., Veettil S.K., Wong P.S., Divya G., Ching S.M., Menon R.K. (2020). Efficacy and safety of posaconazole for the prevention of invasive fungal infections in immunocompromised patients: A systematic review with meta-analysis and trial sequential analysis. Sci. Rep..

[11] Chen L., Krekels E.H.J., Verweij P.E., Buil J.B., Knibbe C.A.J., Brüggemann R.J.M. (2020). Pharmacokinetics and pharmacodynamics of posaconazole. Drugs.

[12] Drugbank Online. https://go.drugbank.com/drugs/DB01263.

[13] Zhao Y., Shen W., Chen Z., Wu T. (2016). Freeze-thaw induced gelation of alginates. Carbohydr. Polym..

[14] Potaś J., Szymańska E., Winnicka K. (2020). Challenges in developing of chitosan–based polyelectrolyte complexes as a platform for mucosal and skin drug delivery. Eur. Polym. J..

[15] Hermanto D., Mudasir M., Siswanta D., Kuswandi B., Ismillayli N. (2019). Polyelectrolyte complex (PEC) of the alginate-chitosan membrane for immobilizing urease. J. Math. Fund. Sci..

[16] Mei J.Y., Huang T., Bai C.H., Fu Z. (2021). Influences of chitosan on freeze–thaw stability of *Arenga pinnata* starch. Int. J. Food Sci. Technol..

[17] Parente M.E., Ochoa Andrade A., Ares G., Russo F., Jiménez-Kairuz Á. (2015). Bioadhesive hydrogels for cosmetic applications. Int. J. Cosmet. Sci..

[18] The United States Pharmacopeia (2016). The United States Pharmacopeial Convention.

[19] Ali S.M., Yosipovitch G. (2013). Skin pH: From basic science to basic skin care. Acta Derm. Venereol..

[20] Gorzelanny C., Mess C., Schneider S.W., Huck V., Brandner J.M. (2020). Skin barriers in dermal drug delivery: Which barriers have to be overcome and how can we measure them?. Pharmaceutics.

[21] Xiong Y., Zhang X., Ma X., Wang W., Yan F., Zhao X., Chu X., Xu W., Sun C. (2021). A review of the properties and applications of bioadhesive hydrogels. Polym. Chem..

[22] Jain A., Gulbake A., Shilpi S., Jain A., Hurkat P., Jain S.K. (2013). A new horizon in modifications of chitosan: Syntheses and applications. Crit. Rev. Ther. Drug Carrier Syst..

[23] Ways M.T.M., Lau W.M., Khutoryanskiy V.V. (2018). Chitosan and its derivatives for application in mucoadhesive drug delivery systems. Polymers.

[24] Kopplin G., Lervik A., Draget K.I., Aachmann F.L. (2021). Alginate gels crosslinked with chitosan oligomers—A systematic investigation into alginate block structure and chitosan oligomer interaction. RSC Adv..

[25] Harrison I.P., Spada F. (2018). Hydrogels for atopic dermatitis and wound management: A superior drug delivery vehicle. Pharmaceutics.

[26] Chavda H., Modhia I., Mehta A., Patel R., Patel C. (2013). Development of bioadhesive chitosan superporous hydrogel composite particles based intestinal drug delivery system. Biomed. Res. Int..

[27] Wróblewska M., Szymańska E., Szekalska M., Winnicka K. (2020). Different types of gel carriers as metronidazole delivery systems to the oral mucosa. Polymers.

[28] Blanco-López M., González-Garcinuño Á., Tabernero A., Del Valle M.E.M. (2021). Steady and oscillatory shear flow behavior of different polysaccharides with Laponite^®^. Polymers.

[29] Carvalho F.C., Calixto G., Hatakeyama I.N., Luz G.M., Gremião M.P., Chorilli M. (2013). Rheological, mechanical, and bioadhesive behavior of hydrogels to optimize skin delivery systems. Drug Dev. Ind. Pharm..

[30] Petrova V.A., Elokhovskiy V.Y., Raik S.V., Poshina D.N., Romanov D.P., Skorik (2019). Y.A. Alginate gel reinforcement with chitin nanowhiskers modulates rheological properties and drug release profile. Biomolecules.

[31] Bu Y., Pandit A. (2022). Cohesion mechanisms for bioadhesives. Bioact. Mater..

[32] Hurler J., Engesland A., Kermany B.P., Škalko-Basnet N. (2012). Improved texture analysis for hydrogel characterization: Gel cohesiveness, adhesiveness, and hardness. J. Appl. Polym. Sci..

[33] Ilić T., Pantelić I., Savić S. (2021). The implications of regulatory framework for topical semisolid drug products: From critical quality and performance attributes towards establishing bioequivalence. Pharmaceutics.

[34] Rençber S., Cheaburu-Yilmaz C.N., Köse F.A., Karavana S.Y., Yilmaz O. (2019). Preparation and characterization of alginate and chitosan IPC based gel formulation for mucosal application. Cellulose Chem. Technol..

[35] Sezer A.D., Cevher E., Hatipoğlu F., Oğurtan Z., Baş A.L., Akbuğa J. (2008). Preparation of fucoidan-chitosan hydrogel and its application as burn healing accelerator on rabbits. Biol. Pharm. Bull..

[36] Vigata M., Meinert C., Hutmacher D.W., Bock N. (2020). Hydrogels as drug delivery systems: A review of current characterization and evaluation techniques. Pharmaceutics.

[37] Bhutani U., Laha A., Mitra K., Majumdar S. (2016). Sodium alginate and gelatin hydrogels: Viscosity effect on hydrophobic drug release. Mater. Lett..

[38] Dash S., Murthy P.N., Nath L., Chowdhury P. (2010). Kinetic modelling on drug release from controlled drug delivery systems. Acta Pol. Pharm. Drug Res..

[39] Nikolova D., Simeonov M., Tzachev C., Apostolov A., Christov L., Vassileva E. (2021). Polyelectrolyte complexes of chitosan and sodium alginate as a drug delivery system for diclofenac sodium. Polym. Int..

[40] European Medicines Agency (2010). Investigation of Bioequivalence.

[41] Shirkhorshidi A.S., Aghabozorgi S., Wah T.Y. (2015). A comparison study on similarity and dissimilarity measures in clustering continuous data. PLoS ONE.

[42] Abd-Elghany M., Klapötke T.M. (2018). A review on differential scanning calorimetry technique and its importance in the field of energetic materials. Phys. Sci. Rev..

[43] Soares J.P., Santos J.E., Chierice G.O., Cavalheiro E.T.G. (2004). Thermal behavior of alginic acid and its sodium salt. Eclética Química.

[44] Mucha M., Pawlak A. (2005). Thermal analysis of chitosan and its blends. Thermochim. Acta.

[45] Dudek G., Turczyn R. (2018). New type of alginate/chitosan microparticle membranes for highly efficient pervaporative dehydration of ethanol. RSC Adv..

[46] Gubanova G.N., Petrova V.A., Kononova S.V., Popova E.N., Smirnova V.E., Bugrov A.N., Klechkovskaya V.V., Skorik Y.A. (2021). Thermal properties and structural features of multilayer films based on chitosan and anionic polysaccharides. Biomolecules.

[47] Dumitriu R.P., Profire L., Nita L.E., Dragostin O.M., Ghetu N., Pieptu D., Vasile C. (2015). Sulfadiazine-chitosan conjugates and their polyelectrolyte complexes with hyaluronate destined to the management of burn wounds. Materials.

[48] Carneiro-da-Cunha M.G., Cerqueira M.A., Souza B.W.S., Carvalho S., Quintas M.A.C., Teixeira J.A., Vicente A.A. (2010). Physical and thermal properties of a chitosan/alginate nanolayered PET film. Carbohydr. Polym..

[49] Pendekal M.S., Tegginamat P.K. (2013). Hybrid drug delivery system for oropharyngeal, cervical and colorectal cancer-in vitro and in vivo evaluation. Saudi Pharm. J..

[50] Spadari C.C., Lopes L.B., Ishida K. (2017). Potential use of alginate-based carriers as antifungal delivery system. Front. Microbiol..

[51] Pritchard M.F., Powell L.C., Jack A.A., Powell K., Beck K., Florance H., Forton J., Rye P.D., Dessen A., Hill K.E. (2017). A low-molecular-weight alginate oligosaccharide disrupts Pseudomonal microcolony formation and enhances antibiotic effectiveness. Antimicrob. Agents Chemother..

[52] Tøndervik A., Sletta H., Klinkenberg G., Emanuel C., Powell L.C., Pritchard M.F., Khan S., Craine K.M., Onsøyen E., Rye P.D. (2014). Alginate oligosaccharides inhibit fungal cell growth and potentiate the activity of antifungals against *Candida* and *Aspergillus* spp.. PLoS ONE.

[53] Perinelli D.R., Campana R., Skouras A., Bonacucina G., Cespi M., Mastrotto F., Baffone W., Casettari L. (2018). Chitosan loaded into a hydrogel delivery system as a strategy to treat vaginal co-infection. Pharmaceutics.

[54] Szekalska M., Wróblewska M., Trofimiuk M., Basa A., Winnicka K. (2019). Alginate oligosaccharides affect mechanical properties and antifungal activity of alginate buccal films with posaconazole. Mar. Drugs..

[55] Peña A., Sánchez N.S., Calahorra M. (2013). Effects of chitosan on *Candida albicans*: Conditions for its antifungal activity. BioMed Res. Int..

[56] Kulig D., Zimoch-Korzycka A., Jarmoluk A., Marycz K. (2016). Study on alginate–chitosan complex formed with different polymers ratio. Polymers.

[57] Costa M.J., Marques A.M., Pastrana L.M., Teixeira J.A., Sillankorva S.M., Cerqueira M.A. (2018). Physicochemical properties of alginate-based films: Effect of ionic cross-linking and mannuronic and guluronic acid ratio. Food Hydrocoll..

[58] Costa P., Sousa Lobo J.M. (2001). Modeling and comparison of dissolution profiles. Eur. J. Pharm. Sci..

[59] Lee J.A., Chee H.Y. (2010). In vitro antifungal activity of equol against *Candida albicans*. Mycobiology.

